# National Cultural Frames and Muslims’ Economic Incorporation: A Comparison of France and Canada

**DOI:** 10.1177/01979183211035725

**Published:** 2022-01-10

**Authors:** Jeffrey G. Reitz, Emily Laxer, Patrick Simon

**Affiliations:** Munk School of Global Affairs and Public Policy, 7938 University of Toronto, Toronto, Canada; 7938 Glendon College, York University, Toronto, Canada; Institut National d'Etudes Démographiques, Paris, France

**Keywords:** Muslims, economic integration, comparative

## Abstract

This article shows that differences in the economic incorporation of Muslims and other immigrant minorities in France and in Canada are mainly related to immigrant selectivity, labor market structures, and welfare transfers. Differences in ethno-specific penalties due to national cultural frames — related to multiculturalism in Canada and secular republicanism in France — are small, affect only the second generation, and are related both to minority household patterns and to treatment in mainstream institutions. Using data on household incomes from two large-scale surveys (Trajectories and Origins in France 2008–2009 and the Canadian National Household Survey 2011) and taking account of cross-setting differences in Muslim and other minority origins, we model cross-generational economic trajectories reflecting the impact of immigrant selectivity, labor market structures, and welfare transfers. Within this framework, we examine four ways that cultural frames may affect minority economic disadvantage: the significance of religion relative to race, citizenship access, labor market discrimination, and minority household patterns, including employment of women in couples and intergenerational cohabitation. Across all minorities, we find a striking cross-national difference in intergenerational economic trajectories: flat in France and upward in Canada, plausibly reflecting institutional differences. Net of sociodemographic controls, both religion and race matter in each setting, and net Muslim disadvantage is similar in each. Citizenship differences have little impact. Labor market earnings discrimination appears similar. A small potential effect of cultural frames appears in second-generation Muslim households: in France, lower female employment rates reduce household incomes, while in English-speaking Canada, more frequent cohabitation with more affluent parents increases household incomes. Yet even these findings do not necessarily diminish the overriding significance of immigrant selectivity, labor market structure, and welfare transfers.

## Introduction

Politicians across Europe have heralded the “retreat” of multiculturalism, but multicultural policies there continue to gain ground (Banting and Kymlicka 2015), albeit with substantial cross-national variations. Some scholars suggest that multicultural policies, as opposed to assimilationist approaches, positively affect newcomers’ incorporation, indicated by rates of naturalization and interracial marriages (Kymlicka 1998, 17–22). Others argue that such policies have limited significance for minority incorporation (van Reekum, Duyvendak, and Bertossi 2012). A review of data on a variety of immigrant outcomes in North America and Europe (Alba and Foner 2015) indicates that more evidence is needed to fully assess multiculturalism's role.

The concept of national “cultural frames” (Small, Harding, and Lamont 2010, esp. 14–20; Lamont et al. 2016) provides a useful approach to assessing multiculturalism's impacts on incorporation. Adapted to issues of immigration and diversity, cultural frames may be understood as encompassing countries’ distinct policy models and the full array of institutional, representational, cultural, and political underpinnings of approaches to migrant selection and settlement, including related traditions, discourses, and ways of thinking about immigration and ethnoreligious diversity in public and private institutions. Such cultural frames are shaped by historical experiences and national cultures and, in turn, may shape the social, political, and economic aspects of minorities’ incorporation.

This article compares a key indicator — minorities’ economic integration — in two countries representing extreme contrasts in the cultural framing of immigration and ethnocultural diversity: France and Canada. In such contrasting cases, the effects of cultural framing might be expected to manifest themselves most clearly, yet our previous work comparing France and Canada with respect to minority social inclusion and political incorporation (Reitz, Simon, and Laxer 2017; Laxer, Reitz, and Simon 2020) found only one such effect: that of citizenship law on naturalization rates (see also Reitz, Simon, and Laxer 2017; Joly and Reitz 2018). Here, we ask about the possible effects of cultural framing on the economic well-being of immigrant minorities.

Throughout its extensive immigration history, France has remained committed to “republican universalism” and the equality of citizens, a tradition relegating cultural differences tied to migration to the private sphere and severely repressing public use of regional languages (Laborde 2008; Hollifield 2014). The French approach to equality is grounded in colorblindness, and references to race in public speeches or statistics are prohibited (Simon 2008). France's emphasis on secularism has been extensively cited in recent debates on regulating religious (mainly Islamic) signs in public and occupational settings (Bowen 2007; Hajjat and Mohammed 2013; Laxer 2018, 2019). By contrast, Canada defines itself as a nation of immigrants and, with other settler societies such as the United States and Australia, prioritizes rapid progress to citizenship (Reitz 2014). Legal rights of racial and religious minorities advanced from the early post-war period in Canada, which is known for its early and steadfast endorsement of multiculturalism and official recognition and support of minority cultures (Kelley and Trebilcock 2010). Increased immigration from non-European origins in the 1970s focused Canadian attention on racial disadvantage, with impacts on policies for multiculturalism and employment equality (Kymlicka 1998).

The primarily French-speaking province of Québec differs from the rest of Canada, which, for convenience (and acknowledging the inaccuracy), we call “English Canada.” Although sharing Canada's immigrant-settlement tradition, Québec's intercultural model of incorporation features a more limited promotion of cultural and religious diversity (Bouchard 2011). The province's social welfare regime is also distinct and resembles that of France in many respects (van den Berg et al. 2017). We suggest that Québec represents a hybrid cultural frame consisting of elements from Canada and France.

The cultural framing of immigration in France and Canada extends beyond policy levers of republicanism and multiculturalism to include the very different places immigration and race occupy in their respective histories, political discourses, and identities. In recent years, immigration politics, including regarding Muslim immigrants, have taken a more positive tone in Canada than France and most other immigration countries (Reitz 2011, 2012; Bloemraad 2012). The 2018 Pew Research Center's (2019) Global Attitudes Survey found that 68 percent of Canadians believed that immigrants “make our country stronger,” the highest percentage of 18 countries surveyed; in France, the figure was 56 percent, about average. International surveys such as the World Values Survey consistently show that Quebec stands between France and English Canada in such assessments. Canadians are more likely to view minority cultures as capable of becoming “fully Canadian” than their French comparators, and Canadians outside Québec are more multicultural in their outlook, more frequently expressing a preference for minorities to maintain their cultures (Reitz, Simon, and Laxer 2017, 30–1).

What differences have these distinctions in national cultural frames made to minority groups’ economic welfare? While this question concerns all minority groups, it arguably has more salience for Muslims, who have been the focus of contemporary debates over multiculturalism and other approaches to immigrant integration (Modood 2013). In France, policymakers have responded to the presence of Muslims by stressing secularism as a feature of republicanism, resulting in measures such as banning headscarves in schools in 2004, prohibiting facial coverings in public spaces in 2011, and establishing a principle of religious “neutrality” in workplaces in 2014 (Hennette-Vauchez 2017). Although Canadian public opinion on immigration generally is far more favorable, there is widespread public concern about Muslims’ cultural adaptability, and accommodation of their cultural differences has encountered obstacles (Reitz 2011; Kazemipur 2014). Still, Canada's official multiculturalism should promote greater acceptance of Muslims and Islamic practices, although Québec is a distinct case, with its policies to limit Muslims’ public religious activity, including a 2019 ban on religious symbols in certain public sector jobs (National Assembly of Québec 2019).

To probe the impact of distinct cultural framings of immigration and diversity on Muslims’ economic incorporation in the three settings, we analyze two comparable national surveys: the French Trajectories and Origins (TeO) survey (2008–2009; see Beauchemin, Hamel, and Simon, 2018) and the 2011 Canadian National Household Survey (NHS) (Statistics Canada, 2017). Our primary outcome of interest is “individual-equivalized” household income (household income adjusted for household size) (OECD 2012). Often overlooked, this critical measure of economic well-being includes the entire sampled population, not just those in the labor force, and all sources of income: employment, welfare state redistribution, and extended family contributions.

Our data sources enable us to address two conceptual and methodological challenges. First, to demarcate the effects of cultural frames, we control for differences in the social and origins composition of Muslim and other minority populations. Muslim immigration in France is largely a legacy of French colonialism in North and West Africa (Blanchard, Bancel, and Lemaire 2005); in Canada, the skill selectivity of immigration policy has produced a more diverse Muslim population (Kazemipur 2014). The surveys enable detailed origins comparisons. Second, to distinguish the effects of cultural frames from those of other contextual differences, we construct a model measuring minorities’ cross-generational economic trajectories, considering education and other sociodemographic factors, which arguably reflect the impact of immigrant selectivity, labor market structures, and welfare transfers on economic well-being. The remaining ethnoreligious effects may result from cultural framing.

We distinguish four mechanisms whereby cultural frames might affect minorities’ economic well-being. First, we consider the general significance of religion, as opposed to race, as a determinant of economic disadvantage, given the differing histories of intergroup relations in North America and Europe. Second, we examine the role of citizenship status, generally more accessible to immigrants in North America than in Europe. Third, we consider labor market discrimination as a manifestation of cultural framing, using earnings analysis to compare the conversion of human capital into earnings. Finally, we probe the effects of two aspects of household patterns on minority economic well-being: the labor force participation of women in couples and patterns of intergenerational cohabitation. Our analysis encompasses diverse ways that the cultural framing of immigration and diversity could impact minority economic incorporation in Canada and France.

We begin by reviewing previous research relevant to the comparison of minority economic well-being in France and Canada, indicating how these analyses require an extension to isolate ethnoreligious effects from immigrant characteristics and institutional factors. We, then, offer theoretical rationales for each of the four cultural frame mechanisms mentioned above, before explaining our methods and presenting descriptive information on socioeconomic and cultural characteristics of minorities in France and Canada. Findings are presented in four sections corresponding to the four mechanisms of cultural framing. We end with a discussion of our main conclusions.

## Previous Research on the Economic Well-Being of Immigrant Minorities

Immigrants have a greater overall economic disadvantage in France than Canada. OECD analysis of 28 countries revealed greater gaps in median individual–equivalized household income between immigrants and the native born in France than in Canada and relatively higher poverty rates for immigrants, with the disadvantage especially acute among recent arrivals (OECD 2012, 51–57). In the French case, many scholars emphasize discrimination faced by Muslim minorities (Adida, Laitin, and Valfort 2016; Beauchemin, Hamel, and Simon 2018). However, comparing the extent of ethnic and religious disadvantage in France and Canada requires consideration of the composition of minority populations. France's legacy of postcolonial immigration and Canada's skill-selective program create important differences in immigrants’ socioeconomic profiles; thus, the analysis must consider the experiences of comparable groups, appropriately matched in terms of human capital and cultural origins.

This analytic task is further complicated by the differing labor market and welfare regimes in each country (Esping-Anderson 1990; Bommes and Geddes 2000; Alba and Foner 2015). Although immigrants generally experience disadvantage integrating into labor markets in many places, labor market theory suggests that liberal labor market regimes, such as those in the United States and Canada, offer easier employment access, though often at low wages, while continental Europe's more regulated labor markets offer better wages and greater job security but create barriers to employment (Reitz et al. 1999; Kogan 2006; Simon and Steichen 2014; Alba and Foner 2015, 11, 62). More generous European social welfare benefits may also moderate poverty for immigrant families (Esping-Anderson 1990).

Taking account of differences in immigrant populations’ socioeconomic profile *and* country-specific labor market structures and welfare state policies may isolate the effects of cultural frames on economic incorporation. Based on comparable government data sources, scholars have identified the economic impacts on immigrants of US-Canada differences in labor markets and related institutions (e.g., Reitz 1998; Borjas 1999; Attewell, Kasinitz, and Dunn 2010). But, as Alba and Foner (2015) show, data limitations preclude similarly detailed comparisons for individual European countries versus either the United States or Canada (Heath and Cheung 2007). Alba and Foner's data allowed them to compare overall household incomes and risk of poverty aggregated across immigrant minorities in the United States, France, and three other European countries, with no controls for human capital or length of residence (Alba and Foner 2015, 62). Within Europe, cross-national comparisons of Muslims’ economic disadvantage are limited to labor force participation or employment, only one component of overall economic well-being (e.g., van Tubergen, Maas, and Flap 2004; Koenig, Mieke, and Güveli 2016; Koopmans 2016).

A few comprehensive trans-Atlantic comparisons of immigrants’ economic status are available. Model and Lin (2002), for example, used 1990s census and survey data to determine whether the economic “cost of not being Christian” was greater in Canada than in the United Kingdom. Based on Portes and Rumbaut's (2006), three “contexts of reception” (government policy, labor market conditions, and ethnic community characteristics), they expected offsetting differences in employment opportunity and earnings, but after controls for human capital, the differences were minimal. Connor and Koenig (2013) used the US General Social Survey, European Social Survey, and Ethnic Diversity Survey to compare the impact of minority (non-Christian) religious affiliation on occupational status in Europe, the United States, and Canada, finding penalties only in Europe and Canada and only for first-generation immigrants and, thus, suggesting that different minorities may experience disadvantage in different contexts. Reitz et al. (1999) used the German Socio-Economic Panel and the 1986 Canadian census to compare the employment earnings of immigrants in Canada and Germany: German education and labor market institutions resulted in higher incomes for low-skill immigrants. However, earnings mobility appeared greater in Canada for immigrants across cohorts, and minority-specific differences were reduced but more variable across origins groups.

We extend these analyses by distinguishing Muslims and other minorities in France and Canada from the mainstream population, focusing on overall economic well-being and employment earnings, and including both first- and second-generation immigrants. Our analysis sheds light on the impact of institutional differences on the economic trajectories of Muslims and other minorities and then addresses the significance of cultural framing.

## Cultural Framing of Immigration and Diversity: An Analytic Agenda

We consider four mechanisms through which the cultural framing of immigration and diversity may affect Muslims’ economic well-being in France and Canada.

*Salience of religion versus race.* Research on Muslims’ incorporation in Europe and North America increasingly emphasizes the interwoven impacts of racialization and attitudes to religion (Meer 2013). The concept of “Islamophobia” captures this joint construction of race and religion, and empirical evidence of its effects is mounting (Lorcerie 2011; Amiraux 2012; Wilkins-Laflamme 2018). Although we concur that race and religion cannot always be analytically separated, we follow others (Zolberg and Woon 1999) in suggesting the two can generate different degrees of hostility and inequality in differing national contexts.

In a qualitative comparison of minority relations in France and the United States, Lamont (2002) concluded that, for historical reasons, race matters more to Americans, while religion is a comparable social divide in France (see Alba and Foner 2015, 117). In France, the reactivation of racist representations shaped during colonial times infuses contemporary hostility to Muslims and to postcolonial minorities more broadly (Blanchard, Bancel, and Lemaire 2005). Lamont's comparison has relevance for Canada as well: while slavery's legacy is far less important in Canada than in the United States, both were shaped by a British colonial system in which slavery flourished (Winks 1997; Whitfield 2016). Contemporary comparisons of the United States and Canada find comparable employment discrimination against blacks and other racial minorities (e.g., Attewell, Kasinitz, and Dunn 2010).

*Access to citizenship.* Ease of access to citizenship is a feature of settler societies, a symbol of inclusion conferring social and economic eligibility (Bauböck 2006). In Canada, while the difference between citizens and permanent residents in formal eligibility for social benefits has eroded, citizenship retains considerable symbolic significance, possibly manifesting in social and economic outcomes (DeVoretz and Pivenko 2005). In France, citizenship eligibility requirements are more stringent, with a residence requirement of five years, but the status of “citizen” may not carry more benefits than in Canada (Hollifield 2014). The citizenship requirement in public employment in France certainly reduces newcomers’ employment opportunities, especially since a quarter of the French labor market consists of public jobs (GELD 2000; Slama 2014). Yet Connor and Koenig (2013, 29) found that citizenship boosted occupational status to a similar extent across all immigrant groups in Europe and Canada, net of other variables, and helped explain religious group disadvantage.

*Discrimination in employment.* Both multiculturalism and assimilationism disavow discrimination based on origins, religion, or race, but their prescribed solutions for discrimination differ (Joppke 2007). Canadian multiculturalism tackles discrimination through tolerance or celebration of difference, while other policies proactively combat discrimination (Kymlicka 1998; Reitz 2014). French republican universalism and secularism aim to attenuate discrimination by promoting the invisibility of differences, leading to antidiscrimination policies that lack the proactive element found in Canada (Simon 2008). Previously, we found similar degrees of reported discrimination among Muslims in France and Canada (Reitz, Simon, and Laxer 2017). Here, we ask whether national cultural frames produce variations in actual employment opportunity by analyzing earnings separately by gender. Our interest in the fate of Muslims is heightened by French legislation enforcing religious neutrality in the public sphere, including workplaces (Laxer 2018; Pauti 2019).

Analyses of discrimination must consider the possible effects of France's institutional environment on employer behavior. Discriminatory decisions might reflect the social welfare regime's influence on employment and social benefits (Algan et al. 2010). In a robust welfare state, more is at stake for employers making new hires, and perceptions of newcomers as deserving or qualified may depend on the extent to which they appear to belong to the national community (Brochmann 2015). In France's labor market system, employers may be more reluctant to hire immigrants and undertake a longer commitment to them, especially if cultural conformity is expected. Meanwhile, employers in Canada's liberal market regime may make short-term commitments to immigrants more easily if the expectation for cultural conformity is less strong.

*Household patterns.* A final factor to consider is whether a minority group's patterns of household resource-sharing differ from those of the mainstream. A crucial component of such resource-sharing is gender and dynamics between adult partners. Addressing the impacts of cultural framing on gendered household arrangements, some suggest that multiculturalism legitimizes traditional cultures that reinforce patriarchal thinking (Okin 1999), while headscarf bans in France have been justified as combatting such thinking (Hennette-Vauchez 2017). Household patterns also have economic effects, either by encouraging women to focus on domestic tasks, rather than entering the labor force, or by valuing their paid work at a lower rate (Meurs and Pailhé 2008). We are interested in tracing the impacts of gendered differences in labor force participation among married couples on minority groups’ relative household incomes. Do such differences represent the impact of cultural framing?

Another component of resource sharing in households is intergenerational sharing in the context of extended family. Research has found ethnic and racial differences in the United States in extended family coresidence, with considerable disparities in the economic contributions to household incomes (Reyes 2018; see also Baland et al. 2016). We are interested in possible differences across settings in the prevalence of intergenerational coresidence among minority groups, as such differences may reflect the retention of cultural traits possibly impacted by national cultural frames, with implications for economic well-being.

## Data and Methods

Our data come from the 2008–2009 French TeO survey and the 2011 Canadian NHS. TeO comprised over 21,000 interviews, including oversampling of immigrants and minority populations. The NHS, conducted on the scale of a 20 percent census but still a voluntary survey like the TeO, had 6,719,000 respondents: 1,604,000 in Québec and 5,115,000 in English Canada. Completed within three years of each other, these surveys capture comparable economic conditions (as a robustness test, we replicated our Canadian analysis in the 2001 census, with similar results). We restrict the analysis to adults aged 25–60 to focus on those with completed education and capable of independence, though not retired. To facilitate analysis of households, we exclude same-sex couples and couples living with parents, about 1 percent in both countries.

We compare the “mainstream” population, comprising respondents born in the country to two native-born parents, to “minorities,” comprising immigrants born outside the country, and to the second generation, comprising those born in the country to at least one foreign-born parent. We further distinguish long-term immigrants, in the country 10 years or more, from more recent arrivals. In Québec, the “mainstream” includes Francophones and Anglophones, and we focus on the former, who are dominant demographically and politically (Laxer 2019).

Immigrant and second-generation minorities in our analysis include Muslims, non-Muslims of non-European origins, and non-Muslims of European origins. The religion question in both surveys taps religious identity (France: “do you currently have a religion,” and if yes, “which one?”; Canada: where all household members’ responses are on one form, “what is this person's religion?”). Non-Muslim includes all other respondents, religiously affiliated or not. We base our assessment of origin on birth region for immigrants and parents’ birth region for second-generation respondents. For the few second-generation respondents with parents born in different regions outside the country, we use the father's birth region. European-origin Muslims are few in both France and Canada and are excluded from the analysis.

The origins of Muslims and other minorities differ across settings. As Table 1 shows, most French Muslims have origins in North Africa, with significant numbers from Turkey and sub-Saharan Africa. France's non-Muslim non-European minorities include many from overseas departments (DOMs), East and Southeast Asia, and Sub-Saharan African (76 percent of West Africans define themselves as Muslims versus 7 percent of Central Africans). In Canada, the main sources are Asia and Europe; Muslims represent a small minority, mainly from Pakistan and the Middle East, with small numbers from North Africa concentrated in Québec.

**Table 1.** Origins of Minorities, Overall and Muslim, Immigrant, and Second Generation, France (2008), English Canada, and Québec (2011).

	France				English Canada				Québec			
	All Minorities		Muslims Only		All Minorities		Muslims Only		All Minorities		Muslims Only	
	First Generation	Second Generation	First Generation	Second Generation	First Generation	Second Generation	First Generation	Second Generation	First Generation	Second Generation	First Generation	Second Generation
Origin												
Caribbean	1.1	0.5	0.0	0.1	4.6	3.9	0.8	3.1	10.1	9.4	0.3	2.8
Central/South America					7.0	3.2	2.2	5.2	11.7	4.2	0.2	0.7
Southern Europe	19.4	39.8	1.8	1.1	6.6	18.6	2.8	2.4	6.7	33.5	1.5	1.9
Other Europe	12.0	9.5	0.6	0.4	17.4	49.5	1.3	1.8	18.2	26.5	1.7	1.9
Maghreb	32.9	36.6	70.4	83.3	0.3	0.1	1.8	1.0	12.1	2.8	53.9	22.1
Sub-Saharan Africa^a^	13.7	5.8	10.5	8.0	5.2	1.6	13.6	23.7	6.7	1.7	7.8	8.5
Turkey	5.9	2.0	12.3	5.5	0.4	0.1	2.2	1.2	0.8	0.4	2.5	3.7
Middle East^b^	1.7	0.8	1.1	0.5	3.9	1.5	17.3	24.5	8.6	5.7	13.8	32.9
West/Central Asia	1.7	0.1	1.5	0.0	3.5	0.4	19.4	4.6	2.7	0.5	7.6	4.5
South Asia	2.2	0.1	1.4	0.9	16.3	4.4	36.2	28.0	5.7	2.0	10.0	20.1
East and Southeast Asia	6.3	3.1	0.2	0.3	30.4	8.7	+	+	14.1	5.8	+	+
Other^c^	3.2	1.2	0.2	0.0	4.4	8.1	2.2	4.6	2.5	7.5	0.7	0.9
Total	100.0	100.0	100.0	100.0	100.0	100.0	100.0	100.0	100.0	100.0	100.0	100.0
(*N*, unweighted)	8,456	8,110	3,243	2,356	731,640	504,250	75,370	8,610	131,000	69,590	25,650	1,480
Percent immigrant, second generation	53.8	46.2	63.1	36.9	59.0	41.0	89.5	10.5	65.7	34.3	94.5	5.5
Percent Muslim, among minorities, by generation	39.4	26.8	n/a	n/a	10.8	1.8	n/a	n/a	19.7	2.2	n/a	n/a

*Source:* Trajectories and Origins Survey, 2008 (France), National Household Survey, 2011 (Canada); all percentages based on weighted data (unweighted Ns rounded for Canadian data).

Sample: Respondents aged 18–60, 18–50 for second generation. Canadian data exclude aboriginal Canadians, foreign-born who are not permanent residents, and those born abroad with Canadian citizenship at birth. French data exclude those born outside metropolitan France with French citizenship at birth (e.g., persons of DOM origin).

^a^ All of Africa except Maghreb, Lybia, and Egypt.

^b^ Egypt, Lybia, Bahrain, Iraq, Israel, Jordan, Kuwait, Lebanon, Oman, Palestine/West Bank/Gaza Strip, Qatar, Saudi Arabia, Syria, United Arab Emirates, and Yemen.

^c^ Other North America, Oceania, and Australia.

Table 2 contains descriptive information on other variables for France, English Canada, and Québec, separately for mainstream and minority populations. Our main dependent variable is the “individual-equivalent” household income adjustment (household income divided by the square root of family size) used in OECD studies (2012). This measure, considered an individual characteristic, includes income from all sources, capturing the impact of an individual's own employment, employment of others in the household, any applicable social benefits or income supports, and extended family contributions. Although imperfect (it does not reflect unequal sharing within households), its comprehensiveness makes it a better reflection of overall economic well-being than individual incomes. Household size tends to be higher among minorities, particularly Muslims. However, since differences are similar across settings, household size adjustments have little impact on cross-setting comparisons. We also analyze individual earnings by gender (for those with positive earnings, defined as annual wages and salaries, reported in France as monthly, and multiplied by 12 for equivalence to Canadian annual data) to test for employment discrimination.

**Table 2.** Descriptive Statistics by Origin Group: France, English Canada, and Québec.

	France				English Canada				Québec			
	Mainstream	European	Non- European^a^	Muslim	Mainstream	European	Non- European^a^	Muslim	Mainstream^b^	European	Non- European^a^	Muslim
Origin group (% of total)	73.5	8.9	11.3	6.3	55.2	20.8	21.1	2.9	78.1	8.7	10.1	3.0
Cohort (% of origin group)												
Recent immigrants	0	54.4	48.3	27.6	0	6.0	30.0	44.9	0	16.0	31.5	61.1
Long-term immigrants	0	37.0	41.0	50.8	0	28.7	56.0	49.1	0	29.8	52.6	36.7
Second generation	0	8.6	10.7	21.7	0	65.3	14.0	6.0	0	54.2	15.9	2.2
Household income	36,290€	35,941€	34,785€	25,618€	$103,900	$112,700	$100,800	$87,700	$84,100	$93,400	$75,600	$60,900
Household size	2.8	2.8	2.9	3.7	2.9	3.1	3.8	4.2	2.7	3.0	3.4	3.7
Equivalized Income	22,412€	22,008€	21,348€	14,393€	$61,900	$65,700	$53,000	$43,400	$51,400	$54,700	$42,000	$32,300
Age (mean)	43.0	41.5	40.8	39.0	43.4	44.6	42.0	40.8	43.9	42.7	40.9	39.6
Education (% of origin group)												
No qualifications	10.1	16.7	11.7	29.3	11.8	8.2	10.5	10.7	14.9	8.5	12.6	10.1
Less than high school	13.3	12.5	11.8	15.9	2.9	2.2	1.0	0.9	3.3	1.8	2.0	1.8
High school	31.3	23.1	17.9	17.5	26.9	23.4	20.4	19.0	19.2	17.5	17.5	14.5
Post-sec. vocational	9.6	9.2	9.0	6.6	33.4	32.8	21.7	17.0	38.5	30.6	25.3	19.3
Post-sec. academic	5.6	7.1	10.4	6.8	3.4	4.4	8.2	7.7	4.4	5.9	7.6	10.1
Bachelor's degree	12.7	11	12.7	9.2	15.0	17.7	23.8	23.8	13.6	20.5	20.6	23.2
Grad./medical degree	17.4	20.6	26.6	14.8	6.6	11.4	14.3	20.9	6.1	15.3	14.3	21.0
Urban residence (%)	15.0	26.6	38.5	42.2	15.5	40.5	68.2	63.3	37.3	82.8	87.9	91.1
Gender (% male)	49.2	48.6	47.5	48.4	49.4	49.4	46.8	50.4	49.2	49.8	47.6	53.8
Employment (% positive earnings)	68.6	73.1	69.6	57.5	79.5	78.6	75.7	62.0	79.2	77.3	72.1	60.7
Marital status (% in couple)	72.1	71.8	64.4	71.2	68.7	70.5	68.8	74.4	67.2	68.2	62.5	76.0
% couple with woman employed	46.5	47.0	40.4	28.7	53.7	56.4	53.1	43.2	54.4	55.0	45.0	40.0
% single living with parents	4.2	5.2	4.6	7.7	5.4	6.8	10.1	9.2	4.2	8.0	9.6	5.0
Citizenship (%)	100.0	71.2	86.6	58.6	100.0	81.8	76.7	77.1	100.0	74.7	73.7	35.1
(*N*, unweighted)^c^	(2439)	(4813)	(5396)	(4106)	(1244000)	(62100)	(474100)	(471100)	(587000)	(22600)	(74700)	(67500)

*Source:* Trajectories and Origins Survey, 2008 (France), National Household Survey, 2011 (Canada). Percentages from weighted data, Ns rounded in Canadian data).

Sample: Respondents aged 25–60, 25–50 for second generation, excluding Muslims of European origins, persons in same-sex couples, and persons in couples living with their parents. Canadian data exclude aboriginal Canadians, foreign-born who are not permanent residents, and those born abroad with Canadian citizenship at birth. French data exclude those born outside metropolitan France with French citizenship at birth (e.g., persons of DOM origin).

^a^ Non-European excludes Muslims.

^b^ Francophone mainstream only.

^c^ Ns are maximum figures, Ns for each statistic varies by less than 2%, except earnings and income variables which vary up to 10%.

Overall, Muslims experienced considerable economic disadvantage relative to mainstream populations in France and Canada, including Québec. Equivalized household incomes of Muslims averaged 36 percent below the mainstream in France, 37 percent in Québec, and 30 percent in English Canada. All “visible minorities” (non-European origins) experienced disadvantage, particularly in Canada, but Muslim disadvantage was most extreme. The degree of Muslim economic disadvantage was also roughly consistent across origin groups in France, English Canada, and Québec (Table 3). In France, the large Muslim groups from Maghreb and sub-Saharan Africa had income levels 36 and 41 percent below those of the mainstream, respectively. In English Canada, income levels of large Muslim groups from South Asia and the Middle East/West Asia were 32 percent below the mainstream. In Québec, those from the Middle East were 34 percent below, and the South Asian gap was 42 percent.

**Table 3.** Household Incomes (IE), Muslim, and Non-Muslim by Regions of Origin: France, English Canada, and Québec, Ages 25–60.

	France			English Canada			Québec		
	Household Income (IE)	Difference From Mainstream	N, Unwgt.	Household Income (IE)	Difference From Mainstream	N, Unwgt.	Household Income (IE)	Difference From Mainstream	N, Unwgt.
Mainstream	€22,501	0.00	(2333)	$61,900	0.00	1,244,000	$51,400	0.00	587,500
(English mainstream, Quebec)							$54,100	+0.05	32,400
Muslim, non-European									
Maghreb	€14,395	−0.36	(2378)						
Mid-east/West Asian				$41,900	−0.32	23,400	$33,700	−0.34	4,700
Sub-Saharan African, Black	€13,191	−0.41	(623)						
South Asian				$42,100	−0.32	26,600	$29,700	−0.42	2,300
Other non-European									
Maghreb	€21,387	−0.05	(1250)						
Mid-east/West Asian				$57,000	−0.08	25,100	$51,900	0.01	11,000
South Asia				$52,300	−0.16	94,400	$38,100	−0.26	4,600
Sub-Saharan African, Black	€19,561	−0.13	(1112)	$59,200	−0.04	26,200	$39,900	−0.22	6,400
DOM – Caribbean	€19,124	−0.15	(934)	$51,800	−0.16	38,300	$37,300	−0.27	14,900
East and Southeast Asian	€22,203	−0.01	(1070)	$52,700	−0.15	235,900	$42,400	−0.18	18,900
European	€22,108	−0.02	(4534)	$65,700	0.06	471,100	$54,700	0.06	67,500

*Note:* European-origin Muslims are not included. Means and Ns for Canada rounded to nearest 100.

*Source:* France: TeO 2008–2009; Canada NHS 2011.

Other visible minority groups experienced significant, though less pronounced, disadvantages. In France, non-Muslims from Maghreb and sub-Saharan Africa had incomes 5 and 13 percent below the mainstream, respectively. In English Canada, non-Muslim South Asians had incomes 16 percent below mainstream levels, compared to 32 percent for Muslim South Asians. Non-Muslims from the Middle East/West Asia had incomes 8 percent below the mainstream, compared to 32 percent for Muslims.

Additional variables (Table 2) include age, educational attainment, urban area of residence (metropolitan areas with significant immigrant minority concentration: Toronto and Vancouver in English Canada; Montreal in Québec; Paris, Lyon, and Marseille in France), gender, labor force status (whether nonzero earnings were reported), marital status (whether in a couple), other household composition variables, and citizenship status.

Educational attainment is measured in seven categories defined similarly in French and Canadian data and with a similar relation to incomes: no qualifications, less than secondary school, secondary school, postsecondary vocational, postsecondary academic, bachelor's degree, and higher degree. Because Canada's immigration since the 1970s is more selective on education (Hollifield 2014; Reitz 2014), many more Canadian immigrants of non-European background had university degrees. Among Muslims in France, 24 percent had university degrees, compared to 45 percent in English Canada and 44 percent in Québec. Correspondingly, more Muslims in France had less than a secondary school diploma.

Visible minority immigrants acquired citizenship at a higher rate in Canada, a difference particularly significant for Muslims (Laxer, Reitz, and Simon 2020). The difference seems higher than we might expect based on longer residency requirements in France. Immigrants of European origin in France, who likely had European Union citizenship, had low rates of French citizenship. Marital status refers to whether a respondent was living in the same household with a spouse. In both countries, roughly 70 percent of the sample was in couples; 30 percent lived as singles. The status of living with parents was significant for singles and relatively common for second-generation adult respondents, particularly those of Muslim and non-European origins.

In the mainstream population, the dual-earner pattern was more frequent across Canada (about 80 percent) than France (64 percent), accompanied by lower labor force participation of women than men in France (OECD 2019). Recent immigrants generally had lower-than-mainstream dual-earner rates; Muslims had the lowest rates everywhere but with a significant cross-setting difference. The proportion of Muslims in couples with the woman employed was 10 percent lower than the mainstream in English Canada, 14 percent lower in Quebec, and 18 percent lower in France (Table 2). Reitz, Phan, and Banerjee (2015) found that lower labor force participation among Muslim women in Canada is only partly related to education and reflects cultural differences as well, including greater childcare responsibilities.

Muslim women's greater labor market integration in Canada, particularly in the second generation, is likely not related to childcare availability, which is more subsidized in France. Instead, we suggest the significance of cultural difference across settings, as indicated by mainstream attitudes to Muslim women wearing headscarves in public spaces. In a survey of 16 nations by PEW Research Center (2005, 5), 78 percent of French respondents regarded banning headscarves as a “good idea,” more than 20 percent higher than respondents in other Western European countries surveyed and in marked contrast to Canada. Within Canada, 55 percent in Québec favored a ban and 32 percent in English Canada (authors’ tabulation of PEW's Canadian data, from Environics Institute 2007). In France, Muslim women wearing headscarves are the main target of Islamophobia, according to the CCIF (Collectif Contre l’Islamophobie en France 2019), an organization working against Islamophobia. Of the 676 complaints received in 2018, 70 percent involved women.

Differences in public hostility to the hijab appear to affect the likelihood that Muslim women will wear it and that those who do will be employed. TeO data may be compared to a 2006 Environics survey of Muslims in Canada (Environics Institute 2007). In France, Muslim women less often wore hijabs and showed a sharper decline across generations. About 26 percent of those in France less than 10 years wore headscarves, compared to 23 percent of women in the country longer and 11 percent of the second generation. In Canada, the figures were 42, 33, and 28 percent, respectively (authors’ tabulation; see also Environics Institute 2016).

Wearing the hijab had a stronger negative impact on women's employment in France, where 25 percent of Muslim women wearing headscarves were active in the labor market, compared to 56 percent of those who did not. In Canada, 38 percent of Muslim women wearing headscarves were active in the labor market, compared to 60 percent of those who did not (authors’ tabulation of 2006 Environics survey). Interestingly, despite more strongly negative views on the headscarf in Québec, patterns there mirrored the rest of Canada.

These differences across settings in Muslim women's cultural practices and labor force participation may reflect the impacts of French republican secularism versus Canadian multiculturalism on Muslim economic well-being. Assimilative pressures in France may reduce the hijab's prevalence, while serving as a barrier to labor force participation for women wearing it, thus lowering household incomes. Multicultural tolerance in English Canada may encourage women to wear the hijab and lower barriers to labor force participation, thus raising household incomes. The French scenario seems a textbook case of how assimilation pressures bring minorities into the mainstream but impose a hefty price on the noncompliant, whether such noncompliance arises from religious commitments or “reactive ethnicity” (Portes and Rumbaut 2006; Nagra 2011). The stigmatization of hijab-wearing reduces its prevalence while discouraging economic participation among those who continue the practice.

Adult singles cohabiting with parents, reflecting intergenerational resource-sharing, were more frequent among all immigrant minorities in France and Canada, including among Muslims, compared to the mainstream population (Table 2). Cohabitation with parents was most pronounced for the second generation (Ferrari and Pailhé 2017), and we find that among Muslims, higher proportions of the second generation lived with parents in English Canada and Québec than in France (even after demographic adjustment).

To identify the effects of cultural frames on economic incorporation, we model the economic trajectories of Muslim and other minorities across immigrant cohorts and into the second generation in France and Canada, comparing them to the mainstream in each setting and taking account of group differences in age, education, and urban residence. Trajectories common to all groups were presumed to derive from broad institutional forces, including labor markets and the welfare state, and their accessibility to immigrants. We focused on the distinctive positions of Muslims and other minorities, net of human capital controls.

We specify the regression in a manner analogous to human capital analyses of employment earnings (e.g., Borjas 1999), which normally include educational attainment, age, and urban area of residence as independent variables:

$$\mathrm{EHHI}=\sum \alpha_{\mathrm{ij}}(\mathrm{Minorit}y_{i}{}^{*}\mathrm{Cohor}t_{j})+\beta (\mathrm{Age})+\gamma_{k}(\mathrm{Educatio}n_{k})+\delta (\mathrm{Urban}),$$

where EHHI is equivalized household income, Minority*_i_* 's (*i*  =  1,2,3) are dummy variables for three minorities (Muslim, non-Muslim non-European, and European), and Cohort*_j_* (*j*  =  1,2,3) are dummy variables for recent immigrants, earlier immigrants, and the second generation (reference category is mainstream population). Age is actual age minus 25 (the minimum age in the sample), Education*_k_* (*k* = 1,…, 6) is a series of dummy variables for education levels, with the reference category a middle-level secondary school diploma (“high school” in Canada, “lower secondary education vocational training, CAP-BEP,” in France), and Urban is residence in one of the urban areas designated above.

Human capital factors are included as independent variables because they affect individual employment earnings and many other determinants of household income. Higher educational attainments lead to higher individual earnings and to a residence in households with other highly educated members, with greater employment opportunity. Urban residence is likely to affect the earnings of all employed household members. Finally, in human capital analyses, men and women are considered separately because earnings determinants differ significantly by gender. Because gender plays a different role in household incomes, the model is similar for men and women.

## Findings

### Race, Religion, and Minority Economic Well-Being Across Cohorts and Generations

**Figure 1.**
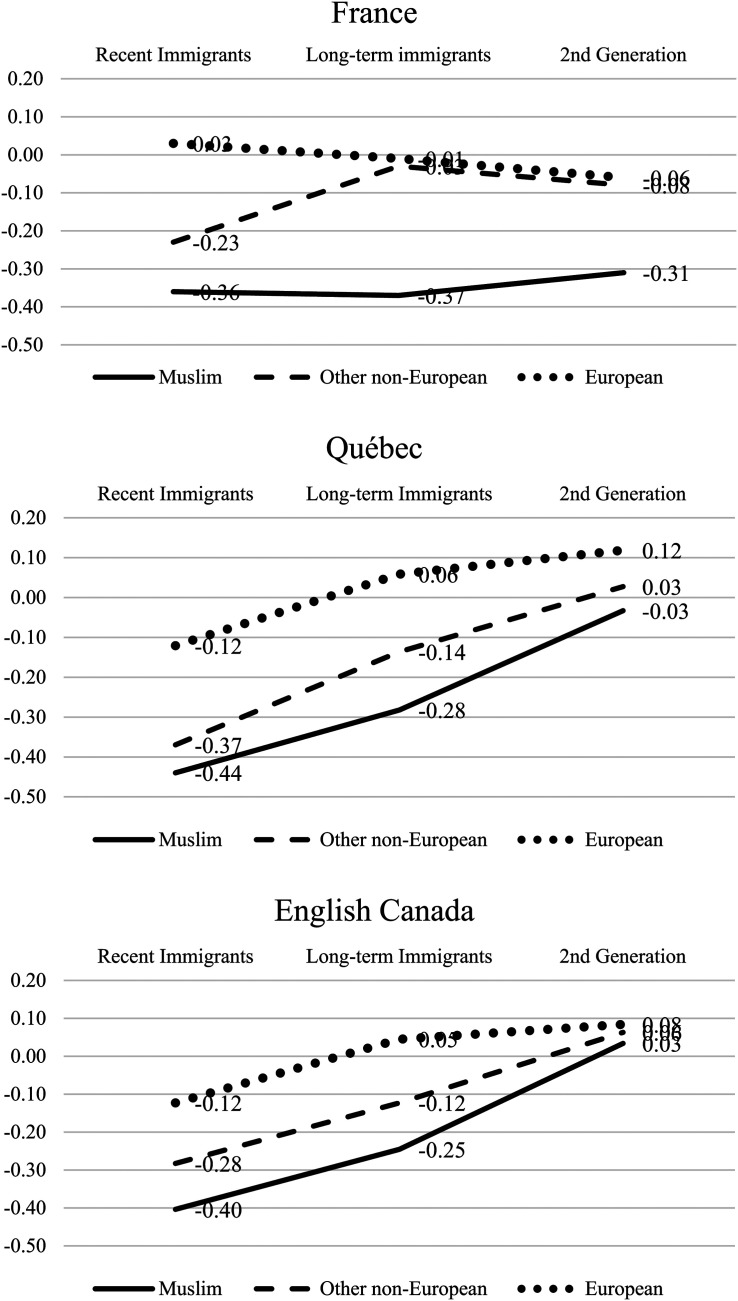
Relative Equivalized Household Incomes by Origins, Immigrant Cohort, Generation, and National Setting (From Table 4, Model 1).

In the analysis of immigrant minorities’ economic well-being by origins and across cohorts and generations, our data show a markedly different pattern in France than in English Canada and Québec (Table 4, Model 1; Figure 1). The overall higher relative incomes in Canada (as seen in OECD data cited above) constitute a complex compositional effect, reflecting the larger size of disadvantaged Muslim minorities in France, particularly those with longer periods of residence, and the smaller proportions there of other non-Europeans and Europeans. In each setting, the relative position of origins groups varies by a period of residence and generation.

**Table 4.** Regressions on Equivalized Household Income, Mainstream, and Minorities by Immigrant-Generation Cohort, Ages 25–60: France, Québec, and English Canada (Figures Are Metric Bs in Local Currency, Significance, and Proportion of Mainstream Values).

	France					
							Québec						English Canada					
	Model 1			Model 2			Model 1			Model			Model 1			Model 2		
	B	Sig.	Prop.	B	Sig.	Prop.	B	Sig.	Prop.	B	Sig.	Prop.	B	Sig.	Prop.	B	Sig.	Prop.
Gen./Origins/Rel. (ref. = mainst.)																		
Mainstream, English (QC only)							2,682	***	0.05	−349	NS	−0.01						
Muslim, non-European																		
Recent immigrant	−8,052	***	−0.36	−6,620	***	−0.29	−22,615	***	−0.44	−31,904	***	−0.62	−24,933	***	−0.40	−34,340	***	−0.55
Long-term immigrant	−8,289	***	−0.37	−7,325	***	−0.33	−14,498	**	−0.28	−21,896	***	−0.43	−15,190	***	−0.25	−22,617	***	−0.37
Second generation	−6,979	***	−0.31	−4,629	***	−0.21	−1,703		−0.03	−8,618	***	−0.17	2,136	*	0.03	−2,169	*	−0.04
Other non-European																		
Recent immigrant	−5,225	***	−0.23	−5,754	***	−0.26	−18,991	***	−0.37	−26,763	***	−0.52	−17,415	***	−0.28	−26,097	***	−0.42
Long-term immigrant	−730	NS	−0.03	−2,950	***	−0.13	−7,002	***	−0.14	−12,158	***	−0.24	−7,566	***	−0.12	−14,326	***	−0.23
Second generation	−1,903	***	−0.08	−2,207	***	−0.10	1,391	**	0.03	−2,940	***	−0.06	3,925	***	0.06	−911	**	−0.01
European																		
Recent immigrant	684	NS	0.03	−1,036	NS	−0.05	−6,245	***	−0.12	−19,029	***	−0.37	−7,594	***	−0.12	−17,527	***	−0.28
Long-term immigrant	−283	NS	−0.01	−1,165	NS	−0.05	2,953	***	0.06	−4,805	***	−0.09	2,808	***	0.05	−3,531	***	−0.06
Second generation	−1,302	*	−0.06	−52	NS	0.00	6,113	***	0.12	944	*	0.02	5,253	***	0.08	1,537	***	0.02
Age (years over 25)				259	***	0.01				482	***	0.01				559	***	0.01
Education (ref. group = sec. school)																		
No qualifications				−2,619	***	−0.12				−10,616	***	−0.21				−10,346	***	−0.17
Less than high school				−1,100	**	−0.05				−4,754	***	−0.09				−4,725	***	−0.08
Postsecondary vocational				2,092	***	0.09				4,930	***	0.10				5,684	***	0.09
Postsecondary academic				3,016	***	0.13				14,573	***	0.28				12,844	***	0.21
Bachelors degree				6,949	***	0.31				23,781	***	0.46				26,004	***	0.42
Graduate/medical degree				12,565	***	0.56				34,875	***	0.68				34,699	***	0.56
Urban residence				3,731	***	0.16				5,048	***	0.10				5,110	***	0.08
Constant	22,510			14,183	***		51,400			22,200	***		61,900			29,700	***	
Unweighted N				15,400						784,300						2,248,220		
R^2^	0.014			0.138			0.012			0.093			0.009			0.056		

*Note:* All origins categories are introduced as dummy variables; other control variables include age, education, and urban residence.

In all Canadian regressions, coefficients are rounded to the nearest integer, the constant is rounded to the nearest 100, and the N is rounded to the nearest 10. Significance levels are: * = *p* < .05, ** = *p* < .01, *** = *p* < .001.

*Sources:* TeO (2008, 09) and NHS (2011).

For Muslims, relative equivalized household incomes for the most recent immigrants are at roughly the same low level across settings: 36 percent below mainstream levels in France, 40 percent below in English Canada, and 44 percent below in Quebec. The low incomes in Canada are surprising, given immigrants’ much higher education levels. At the same time, more generous social benefits in France could set a higher floor for household incomes, regardless of education. In any case, in France, the extent of disadvantage for immigrants in the country longer is much the same (−37 percent); corresponding groups in English Canada and Québec showed substantially better economic outcomes (−25 and −28 percent, respectively). Second-generation Muslims’ relative position becomes even more favorable in English Canada (+3 percent) and Québec (−3 percent) than in France (−31 percent).

Disadvantage of other non-European minorities is significant, though somewhat less than for Muslims, indicating race and religion matter in all three settings. The contrasting France-Canada progression across cohorts and generations seen for Muslims holds to a degree for non-European non-Muslims and is clear for European-origin minorities. The most notable observation is the relatively better position of non-Muslim visible minority immigrants in France, both recent and longer term. In this sense, Muslim religion matters after race is taken into account. Meanwhile, both Québec and English Canada show the greater importance of race than religion.

The Canada-France contrast in economic patterns across cohorts and generations underscores the importance of time-related variables in comparative research on immigrant integration. And the extent to which this contrast applies similarly to all origin groups suggests the significance of broader institutional structures in determining their economic well-being. Whether to interpret the results as deriving from contextual differences in mobility or progress for minority groups over time (“upward mobility” in Canada versus economic “stagnation” in France) requires further analysis, considering variations in the social composition of the respective populations.

Immigrants to Canada had higher education levels overall, but recent immigrants in both countries had more education than earlier arrivals. This shift likely resulted from rising education levels in source countries and, in the case of Canada, the move to increase immigration selection standards in the 1990s. Second-generation Muslims in both Canada and France had higher education levels than immigrants. In France, relatively few had less than secondary qualifications, most had attained postsecondary qualifications, often vocational rather than academic, and many had acquired bachelor's degrees. In Canada, educational mobility meant that more second-generation Muslims had university qualifications.

**Figure 2.**
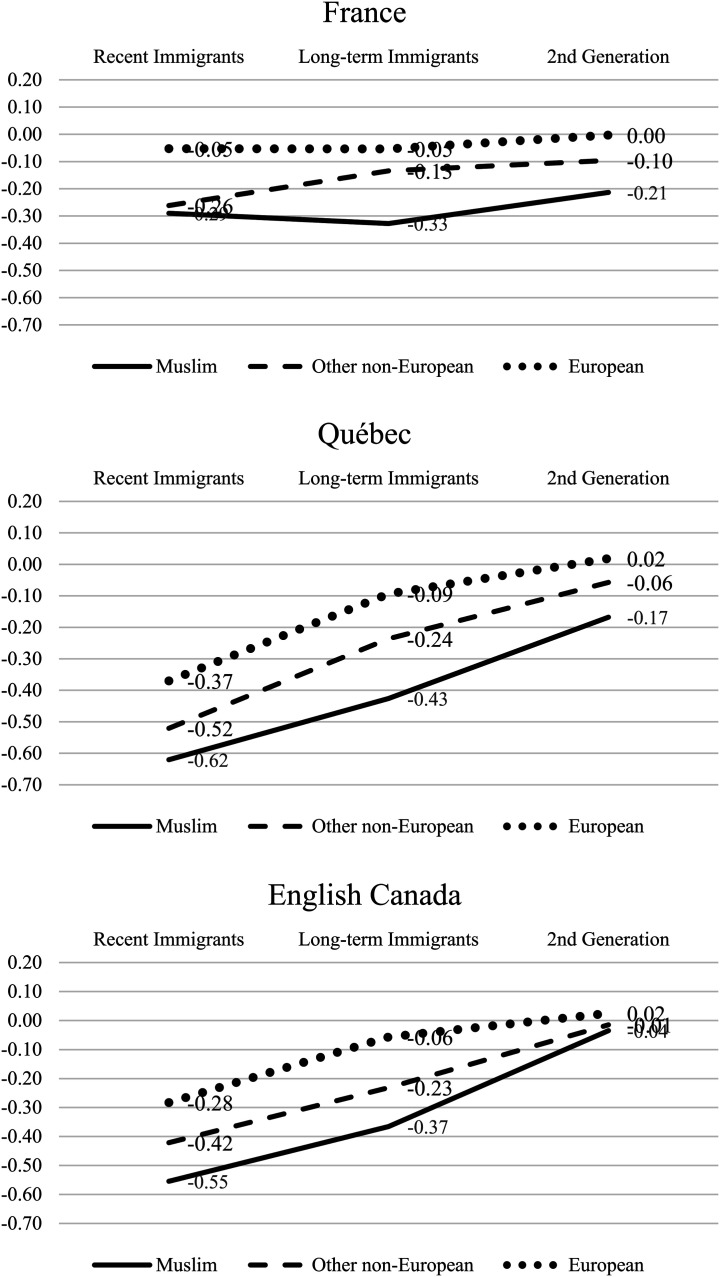
Relative Equivalized Household Incomes by Origins, Immigrant Cohort, Generation, and National Setting, After Controls for Education, Age, and Urban Area (From Table 4, Model 2).

Analysis of incomes net of adjustments for differences in education, age, and urban residence (Table 4, Model 2; Figure 2) finds setting-distinctive patterns across cohorts and generations for all origin groups, suggesting institutionally generated differences in immigrant experience by country. Race and religion created pervasive disadvantages across settings, with interesting variations.

Economic trajectories of all minorities across cohorts and generations show a steep slope for English Canada and Québec but are relatively flat in France. When human capital and other individual characteristics are controlled for, the equivalized household incomes of recent immigrant Muslims and other minorities in Canada were substantially *less* than the corresponding groups in France. New Muslim immigrants’ economic position in France was 29 percent below that of the mainstream; in English Canada, 55 percent below, and in Québec, 62 percent below. Corresponding differences apply to other origins groups. However, whereas in France, penalties in equivalized household income were roughly the same for recent immigrants, earlier immigrants, and the second generation, in Canada, economic penalties were 60 percent for recent immigrants compared to 40 percent for earlier ones. By the second generation, outcomes in Canada were more favorable, with second-generation Muslims in France reporting household incomes 21 percent below the mainstream, compared to 17 percent in Québec and 4 percent in English Canada. In other words, Muslims in Canada enjoyed much better outcomes, the longer they were in the country, particularly outside Québec.

These differences — a stable economic disadvantage for Muslims in France versus more extreme initial inequality followed by a pattern of improved economic integration in Canada — may be related to institutional differences: the more regulated labor markets and robust social redistribution in France than in Canada. In Canada, the immigrant selection system recruits the highly educated, but most immigrants enter the country without prearranged employment, and employers’ lack of recognition of foreign-acquired qualifications causes a prolonged economic struggle for many (Statistics Canada 2005). France's regulatory framework seems to benefit new arrivals, arguably by providing better employment and standard pay levels and offsetting social supports, whereas Canada's more open labor markets provide an opportunity for improvement over time.

Our analysis of French/Canadian differences in economic trajectories reveals broad similarity in the impact of race and religion but with an interesting difference. The relative disadvantages of origins groups were the same in all three settings. Non-European origins minorities encountered more obstacles translating education into economic well-being than European-origin minorities. Among the former, Muslims encountered even more obstacles, suggesting a racial factor and a Muslim religious factor in all three settings.

Notably, the religious penalty encountered by second-generation Muslims was much reduced in English Canada, where Muslim status faded to near insignificance. The more positive economic outcomes for second-generation Muslims in Canada than in France were partly explained by higher educational levels, arguably attributable to the influence of their more highly educated immigrant parents (Chen and Hou 2019). Now we see that for English Canada, there is an important additional difference beyond what is explained by education levels or other demographic variables. To explain this interesting finding, we consider the impact of cultural frames, examining differences in citizenship access, labor market discrimination, and household patterns.

### Economic Impact of Citizenship Access

In France, about one in six recent immigrants in our sample had acquired citizenship versus one in three in Canada. Between half and two-thirds of longer term immigrants in France had citizenship, compared to nearly all longer term immigrants in Canada. Rates were somewhat lower for Muslims in France than for other visible minorities and higher in Canada (immigrants of European origins had low rates of acquiring French citizenship and most likely had EU citizenship). We find that citizenship was significantly related to household incomes in both France and Canada, somewhat more so in Canada. Net of other variables, citizenship boosted incomes by 6 percent in France, 8 percent in English Canada, and 13 percent in Québec (results on citizenship not shown). The extent to which this empirical relation is causal is unclear, as citizenship acquisition may serve as a proxy for characteristics promoting economic success.

We find little economic relevance for differences in citizenship across settings. Citizenship's impact on economic well-being was most significant for recent immigrants; for Muslims, low rates of citizenship acquisition in France accounted for about 5 percent of their income disadvantage, compared to 3 percent for Muslims in English Canada. Hence, after taking account of citizenship differences, the relative earnings of recent arrivals were *higher* in France than in English Canada. In other words, high rates of citizenship acquisition in English Canada are one reason why recent immigrants’ initial incomes were not lower. Québec showed a large income boost associated with citizenship, more than 50 percent higher than in English Canada and more than double the rate in France; lack of citizenship accounted for 7 percent of the relatively low incomes of recent Muslim immigrants in Québec.

### Labor Market Discrimination and Disadvantage

**Figure 3.**
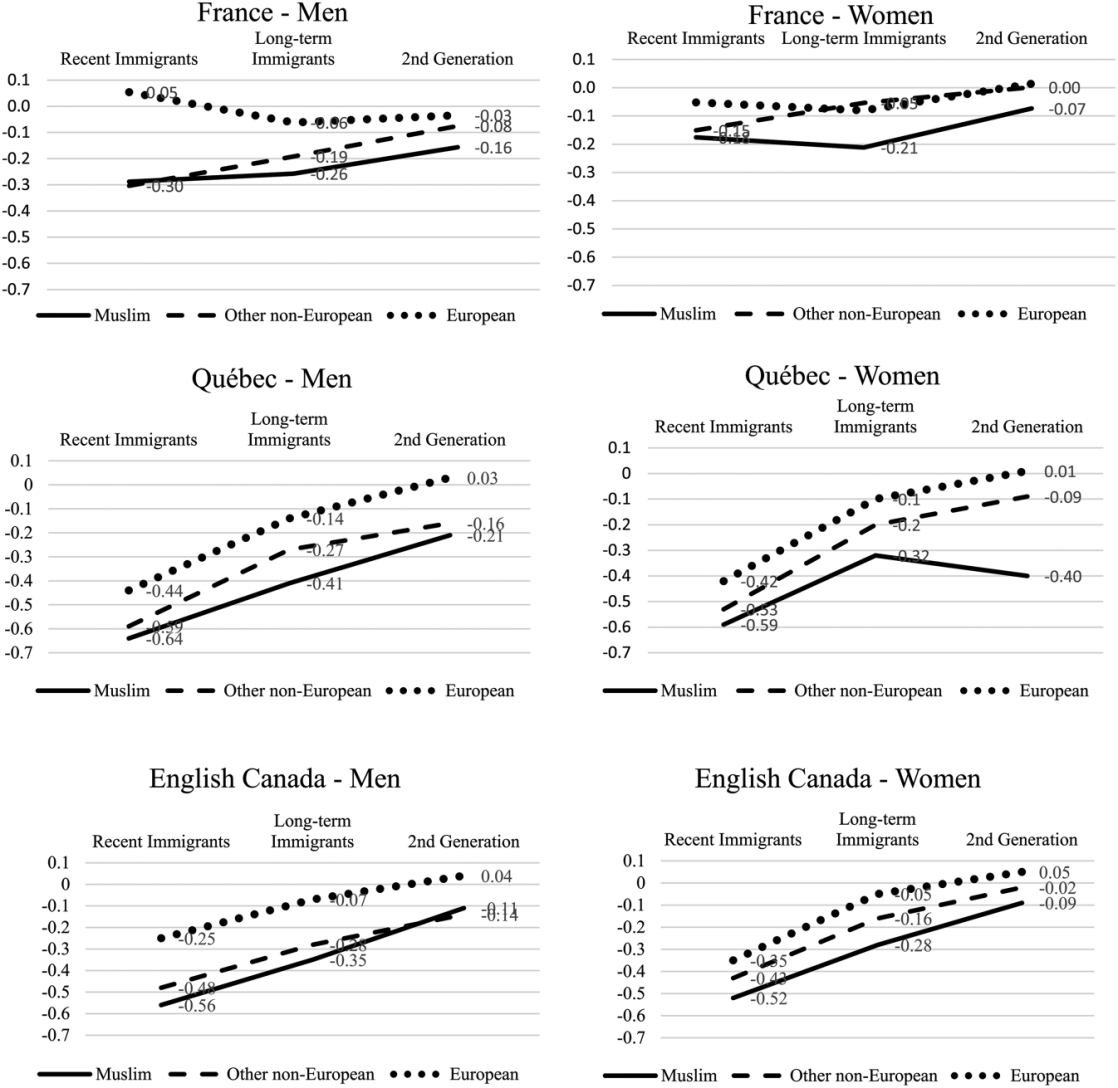
Relative Employment Earnings by Gender, Origins, Immigrant Cohort, Generation, and National Setting, After Controls for Education, Age, and Urban Area (From Table 5).

To determine the extent to which cross-setting differences in Muslims’ household incomes derived from differences in labor market discrimination, we analyzed employment earnings for men and women reporting any positive earnings. For each setting, we presented human capital regressions for minorities relative to the mainstream, by immigration cohort and generation and by gender, controlling for age, education, and urban residence (Table 5; Figure 3). Findings show pervasive racial and religious disadvantage in employment earnings across settings, in many respects mirroring those for equivalized household income. The earnings of recent immigrants, after considering education, age, and urban area, were low in English Canada and Québec, compared to France, a difference affecting all origin groups for both men and women (each compared to the respective mainstream gender). Results were similar for longer term visible minority immigrants, especially Muslims. Citizenship acquisition was significantly related to earnings in each setting (results not shown). The relation was stronger in Canada, particularly in Québec, and relatively greater for women. After adjustment for cross-national differences in citizenship acquisition, recent immigrants showed an even greater disadvantage in Canada, with little difference by origin.

**Table 5.** Regressions on Individual Earnings, for Mainstream and Minority Workers With Positive Earnings, Ages 25–60: France, Québec, and English-Canada; (Figures Are Metric Bs in Local Currency, Significance, and Proportion of Mainstream Values).

	France					
							Québec						English Canada					
	Men			Women			Men			Women			Men			Women		
	Model 1			Model 2			Model 1			Model 2			Model 1			Model 2		
	B	Sig.	Prop.	B	Sig.	Prop.	B	Sig.	Prop.	B	Sig.	Prop.	B	Sig.	Prop.	B	Sig.	Prop.
Gen./Origins/Rel. (ref. = mainst.)																		
Mainstream, English (QC only)							350			−372								
Muslim, non-European																		
Recent immigrant	−7,524	**	−0.29	−3,167	*	−0.18	−32,728	***	−0.64	−21,772	***	−0.59	−35,641	***	−0.56	−21,522	***	−0.52
Long-term immigrant	−6,716	***	−0.26	−3,804	***	−0.21	−20,957	***	−0.41	−12,009	***	−0.32	−21,963	***	−0.35	−11,633	***	−0.28
Second generation	−4,079	NS	−0.16	−1,326	NS	−0.07	−10,497		−0.21	−14,961	***	−0.40	−6,922	***	−0.11	−3,557	**	−0.09
Other non-European																		
Recent immigrant	−7,928	**	−0.30	−2,719	**	−0.15	−30,197	***	−0.59	−19,614	***	−0.53	−30,482	***	−0.48	−17,936	***	−0.43
Long-term immigrant	−5,002	**	−0.19	−970	*	−0.05	−13,958	***	−0.27	−7,253	***	−0.20	−17,490	***	−0.28	−6,872	***	−0.16
Second generation	−1,961	NS	−0.08	32	NS	0.00	−8,334	***	−0.16	−3,278	***	−0.09	−8,909	***	−0.14	−781	**	−0.02
European																		
Recent immigrant	1,414	NS	0.05	−942	NS	−0.05	−22,180	***	−0.44	−15,658	***	−0.42	−15,621	***	−0.25	−14,432	***	−0.35
Long-term immigrant	−1,614	NS	−0.06	−1,450	**	−0.08	−6,947	***	−0.14	−3,885	***	−0.10	−4,720	***	−0.07	−1,931	***	−0.05
Second generation	−890	NS	−0.03	249	NS	0.01	1,556		0.03	334		0.01	2,423	***	0.04	1,921	***	0.05
																	***	
Age (years over 25)	323	***	0.01	265	***	0.01	631	***	0.01	472	***	0.01	786	***	0.01	531	***	0.01
Education (ref. group = sec. sch.)																		
No qualifications	−2,887	**	−0.11	−1,917	***	−0.11	−7,715	***	−0.15	−8,626	***	−0.23	−8,052	***	−0.13	−7,171	***	−0.17
Less than high school	−807	NS	−0.03	−1,812	***	−0.10	−2,731	***	−0.05	−5,122	***	−0.14	−204	NS	−0.00	−3,361	***	−0.08
Postsecondary vocational	1,941	NS	0.07	3,262	***	0.18	6,349	***	0.12	4,941	***	0.13	10,076	***	0.16	5,932	***	0.14
Postsecondary academic	4,506	**	0.17	3,068	***	0.17	17,351	***	0.34	15,359	***	0.41	18,338	***	0.29	14,243	***	0.34
Bachelors degree	6,972	***	0.27	6,031	***	0.34	30,066	***	0.59	23,095	***	0.62	34,177	***	0.54	23,043	***	0.55
Graduate/medical degree	19,344	***	0.74	10,720	***	0.60	47,539	***	0.93	35,831	***	0.97	48,811	***	0.77	32,192	***	0.77
Urban	6,030	***	0.23	3,452	***	0.20	5,835	***	0.11	4,187	***	0.11	3,535	***	0.06	4,502	***	0.11
Constant	14,775	***		9,929	***		12,600	***		6,400	***		18,100	***		9,300	***	
Unweighted N	5,679			5,760			326,900			315,900			926,900			917,700		
R2	0.121			0.319			0.091			0.154			0.053			0.108		
Mean earnings, mainstream	26,129			17,992			50,900			37,100			63,100			41,700		

Significance levels are: * = *p* < .05, ** = *p* < .01, *** = *p* < .001.

*Sources:* TeO (2008, 09) and NHS (2011).

The second generation is of specific interest, given the relatively higher level of household incomes for Muslims in English Canada than France or Québec. Second-generation Muslims’ comparative earnings disadvantage was remarkably similar across settings, with the exception of women in Québec. Among men, the Muslim earnings penalty relative to human capital was somewhat greater in France and Québec than in English Canada (−16, −21, and −11 percent, respectively). For women, it was slightly less in France (−7 percent) than in English Canada (−9 percent) and much greater in Québec (−40 percent; a surprising figure but with a higher standard error). The presence of somewhat less religious discrimination for second-generation Muslim men in English Canada could account for some of their higher household incomes compared to France and Québec, albeit offset by the contrary results for women. Overall, the cross-setting difference in equivalized household income disadvantage for second-generation Muslims (−21 percent in France, −4 percent in English Canada; Table 4, Model 2) is likely only slightly (if at all) due to these groups’ respective labor market earnings or differences in employment discrimination. Across Québec and English Canada, second-generation Muslims’ earnings disadvantage appeared greater than the overall equivalized household income disadvantage, suggesting that other sources of household income might be relevant.

### Household Pattern Effects

We examine two aspects of household composition: for persons in couples, whether the woman was doing paid work outside the home, and for singles, whether the person was living with parents. Table 6 extends Table 4 (to save space, we show effects only as proportions of mainstream incomes). Variables are added in sequence, first a dummy variable for couples (Model 3), then a variable for women working, with minority-group interactions (Model 4), and finally, a variable for singles living with parents, again with minority-group interactions.

**Table 6.** Regressions on Household Income (IE), Mainstream, and Muslim Minorities by Immigrant-Generation Cohort, Ages 25–60: France, Québec, and English Canada, With Controls for Human Capital and Couple Status in Model 3 and Additional Controls for Household Composition in Models 4 and 5. (Coefficients Expressed as Proportions of Mainstream Household Incomes, IE).

	France					
							Quebec						English Canada					
	Model 3		Model 4		Model 5		Model 3		Model 4		Model 5		Model 3		Model 4		Model 5	
	Prop.	Sig.	Prop.	Sig.	Prop.	Sig.	Prop.	Sig.	Prop.	Sig.	Prop.	Sig.	Prop.	Sig.	Prop.	Sig.	Prop.	Sig.
Gen./Origins/Rel. (ref. = mainst.)																		
Mainstream, English (QC only)							0.00	NS	0.00	NS	0.00							
Muslim, non-European																		
Recent immigrant	−0.32	***	−0.26	***	−0.23	***	−0.66	***	−0.55	***	−0.41	***	−0.58	***	−0.51	***	−0.53	***
Long-term immigrant	−0.34	***	−0.30	***	−0.29	***	−0.45	***	−0.37	***	−0.27	***	−0.38	***	−0.33	***	−0.37	***
Second generation	−0.19	***	−0.15	***	−0.15	***	−0.11	*	−0.05	NS	−0.19	*	−0.02	***	0.02	NS	−0.07	***
Other non-European																		
Recent immigrant	−0.25	***	−0.22	***	−0.18	***	−0.54	***	−0.45	***	−0.34	***	−0.44	***	−0.39	***	−0.39	***
Long-term immigrant	−0.12	***	−0.11	***	−0.09	**	−0.24	***	−0.18	***	−0.14	***	−0.24	***	−0.20	***	−0.21	***
Second generation	−0.09	***	−0.08	**	−0.04	NS	0.00	NS	0.05	***	−0.10	***	0.01	**	0.04	***	0.01	NS
European																		
Recent immigrant	−0.07	NS	−0.04	NS	−0.02	NS	−0.41	***	−0.38	***	−0.25	***	−0.32	***	−0.32	***	−0.31	***
Long-term immigrant	−0.06	NS	−0.07	NS	−0.06	NS	−0.10	***	−0.10	***	−0.10	***	−0.06	***	−0.07	***	−0.08	***
Second generation	0.00	NS	0.00	NS	0.01	NS	0.02	**	0.03	**	−0.01	NS	0.03	***	0.02	***	0.01	***
Couple (v. single)	0.19	***	0.08	***	0.14	***	0.27	***	0.07	***	0.12	***	0.20	***	0.04	***	0.10	***
Woman working (for couples)			0.17	***	0.18	***			0.27	***	0.27	***			0.21	***	0.21	***
Woman working — Muslim			−0.05	NS	−0.05	NS			−0.09	***	−0.07	***			−0.05	***	−0.02	NS
Woman working — Other non-Euro.			−0.03	NS	−0.06	NS			−0.10	***	−0.07	***			−0.07	***	−0.06	***
Woman working — European			0.00	NS	−0.01	NS			−0.01	NS	0.00	NS			0.01	**	0.01	**
Woman working — English (Quebec)									0.03	*	0.05	***						
Living with Parents — for singles					0.45	***					0.34	***					0.31	***
Living w. Parents — Muslim					−0.17	NS					0.08	**					0.07	***
Living w. Parents — Other non-Euro.					−0.26	**					0.02	NS					−0.05	***
Living w. Parents — European					−0.07	NS					−0.06	***					−0.07	***
Living w. Parents — English											0.11	***					0.09	***
Unweighted N	15,400						778,070						2,234,990					
R^2^	0.152		0.161		0.172		0.111		0.120		0.125		0.063		0.067		0.071	
																		
Mainstream IEHHI	22,510 €						$51,300						$61,900					

*Note:* Couples living with parents and same-sex couples are not included. All origins categories are introduced as dummy variables; other control variables include age, education, and urban residence. In all Canadian regressions, coefficients were rounded to the nearest integer, the constant is rounded to the nearest 100, and the N is rounded to the nearest 10. Significance levels are: * = *p *< .05, ** = *p *< .01, *** = *p* < .001.

*Sources:* TeO (2008, 09) and NHS (2011).

Across settings, couple status boosted equivalized household incomes. Much of this boost reflects female labor market participation (the couples’ coefficient was dramatically reduced when “women working” was entered), but effects for Muslims and other visible minorities were weaker (interactions with “women working” are negative). The impact of female labor market participation on second-generation Muslims’ relative equivalized household incomes in the cross-setting comparison is negligible (comparing Models 3 and 4). Before controls for women working, the second-generation Muslim disadvantage was 19 percent in France and 2 percent in English Canada. After controls, the cross-setting difference remained 17 percent (15 percent disadvantage in France, 2 percent advantage in English Canada). The difference between France and Québec was less before the control (8 percent) and not at all reduced afterward (10 percent).

Regression results clarify this lack of effect. Although second-generation Muslim women's relative labor force participation in France is significantly lower than in English Canada, by roughly 10 percent (after adjustments for demographics), the impact is less because of minority disadvantage. Muslim women's contribution to household incomes in France was 12 percent, compared to 17 percent for mainstream women (the direct effect). Consequently, raising second-generation Muslim women's labor force participation in France to the English-Canada level would raise household incomes only by 1.2 percent (a 12 percent addition in earnings applied to an increase of 10 percent in women working). Yet other couple-status factors offset even this small effect. France showed a relatively large couple-status effect unrelated to women's employment. Specifically, being in a couple boosted equivalized household incomes by 8 percent whether the woman worked or not. In Québec, the bonus was 7 percent, but in English Canada, it was only 4 percent. This boost may be a product of family benefits and income supports raising Muslim households’ relative incomes, suggesting that France's welfare state could offset the impact of traditional cultural patterns and thereby possibly encourage them.

Singles living with parents had dramatically higher equivalized household incomes (Table 6, Model 5) in all three settings. However, in France, the effects for visible minorities, including Muslims (reflected in the coefficients for interaction terms), were much less positive, while in Canada, the effects for Muslims were actually more positive than for the mainstream (indicated by the minority interaction terms). A more positive impact of parental cohabitation in Canada may reflect the better economic position of those parents. Our data (Table 4) show that immigrants in Canada long enough to have adult children were comparatively well off, mainly because of their better education. In effect, Canada's selective immigration policy impacted the second generation by boosting economic resources available for intergenerational sharing.

After considering the differences in patterns of living with parents, net of individual characteristics, the second-generation Muslim disadvantage was less, as was the difference across settings. Notably, the difference between France and English Canada, previously 17 percent overall, dropped to 8 percent after controlling for singles living with parents. Therefore, the trend toward equity in Muslim household incomes for the second generation in English Canada was strongly affected by the greater prevalence of living with parents. Net of this family contribution, second-generation Muslims’ economic position in Québec appeared more negative and much closer to France. Additional data from the surveys indicate that parents made financial contributions to second-generation households where there was no cohabitation; such contributions were more common in Canada than in France. We cannot compare the amounts, however, which are not in the French data.

## Conclusions

Muslim minorities’ economic struggles in France's *banlieues* are well known and highly visible (Lorcerie 2011; Amiraux 2012). In contrast, Muslims who arrive in Canada as part of a skill-selected immigrant population are often included in the discourse of “Canadian exceptionalism” as exemplars of successful incorporation (Kazemipur 2014, chapter 7). Yet our comparative analysis suggests that these countries’ differing models of immigration and cultural framings of immigration and diversity do not matter much, if at all, in producing differing economic outcomes for Muslims in France versus Canada. In fact, we find substantial religious and ethnoracial disadvantages for minorities, including Muslims, in *both* countries. Most cross-national differences that we do observe are related to minorities’ differing socioeconomic profiles and country-specific labor market structures and welfare state policies. Differences in cultural framing of immigration are potentially relevant only for the second generation, and they are likely quite small, as often related to social patterns within minority households as to treatment by mainstream institutions.

Immigrant minorities’ economic trajectories followed distinct patterns in each country and differed by origin group, race, and religion. In Canada, where immigrants’ educational levels are comparatively high, recent arrivals struggled economically, and longer term immigrants did better. The children of immigrants obtained considerable educational and economic success in Canada, a pattern observed across minority groups and stemming indirectly from immigrant selectivity and immigrant parents’ higher educational levels (Chen and Hou 2019). In France, the immigrant economic disadvantage was moderated for all origins among recent arrivals and longer term immigrants, regardless of low education levels, and the experience of the second generation was similar. The French welfare state likely contributed to this lack of cohort variation, but our data on employment earnings suggest that the differences in labor market processes were equally important. In each setting, race and Muslim status both mattered, imposing additional economic disadvantages within the country-specific pattern.

Alba and Foner (2015, 47–67) argue that poverty wage levels for low-status immigrants in liberal market settings represent “some degree of integration.” Presumably, integration of those earning poverty wages depends on the potential for their upward mobility, and our findings for Canada support the hypothesis that liberal markets create opportunities, despite low initial earnings. Still, the hypothesis might not apply equally in the United States, where greater overall inequality — a result of weaker union influence, among other institutional factors — implies less mobility (Bradbury et al. 2015).

Our analysis of cultural framing considered all minority groups and cohorts, but most attention focused on second-generation Muslims, since in France, this group had poorer relative economic outcomes than their counterparts in Canada. Among the four potential implications of cultural framing, we considered, we find little indication that any of the first three matter much for Muslims. First, while race and religion mattered in both countries, religion mattered somewhat more in France and race more in Canada, arguably reflecting the differing composition of immigrant minorities in each country. In the end, however, overall Muslim disadvantage — combining the race and religion effects — was similar across settings. Second, greater access to citizenship in Canada versus France did not seem to be a major factor affecting relative economic outcomes for minorities. Even if the correlation between citizenship acquisition and economic outcomes is causal, greater ease of citizenship access in Canada was relevant mainly for new arrivals, for whom economic outcomes were particularly poor. For longer term immigrants, citizenship acquisition could have benefitted Muslim immigrants in Canada but would be irrelevant for the second generation where the main cross-national differences in economic outcomes occurred. Third, our analysis produced little evidence of a meaningful France–Canada difference in earnings discrimination based on race or religion, as earnings disadvantages net of human capital seemed comparable across settings. For second-generation minorities, we note a small positive difference for Muslim men, but not women, in English Canada and a larger negative difference for Muslim women in Québec. Discrimination in the labor market, in a context of increasing Islamophobia, appeared pervasive across settings.

Fourth, our concluding analysis of household patterns — manifested by women's employment and intergenerational cohabitation — produced perhaps the most interesting findings. Among second-generation Muslim households, couples with women in paid employment were more often the exception in France, and household incomes were lower as a result. The impact that fewer dual-earner households in France had on the overall France–Canada difference in Muslims’ household incomes was quite small and entirely offset by contributions to household income in France from other sources, possibly government income supports or other social benefits. However, the difference in women's employment may have significance, as evidence of a cultural framing effect. In France, pressures to enforce republican secularism in the public sphere have been manifested in headscarf bans that sometimes fail, or induce “reactive ethnicity,” and Muslim women who wear headscarves are very unlikely to be employed. In Canada, multicultural tolerance, as expressed in more widespread wearing of the headscarf, might favor economic incorporation as evidenced by the weaker negative association between headscarf wearing and employment. In both countries, Muslim economic integration involved women's entry into the labor market with associated economic pressures and household issues. Still, the economic impact of this France–Canada difference is small, and since it is offset by other sources of income, possibly government income supports, it ultimately contributes nothing to explaining the poorer economic position of second-generation Muslim households in France than in English Canada.

Higher rates of parental cohabitation and parents’ financial contributions made an important contribution to the more positive economic well-being for second-generation Muslim singles in Canada than in France. If this Canadian pattern reflects the stronger retention of cultural traditions for intergenerational support and if it could be attributed to Canadian multicultural policy or related popular attitudes, this finding would be the most persuasive demonstration of a cultural framing effect in our results. However, other interpretations are quite plausible. The large positive size of the effect of living with parents in Canada is almost certainly related to the parental generation's greater affluence, which, in turn, is related to Canada's immigrant selectivity. Still, the cultural interpretation, possibly in interaction with the impact of immigrant selectivity, remains a significant possibility.

Even though the different economic trajectories of immigrant minorities in France and Canada are shaped primarily by differences in the composition of immigrant groups and respective national institutional contexts, the small effects of cultural framing on minorities’ economic incorporation command interest because of the large role cultural frames play in national political discourses on immigration. Yet cultural frames for immigration and diversity have their own political dynamic, and based on our findings, that political dynamic would appear to intersect with processes of minorities’ economic integration only in very specific aspects, such as when legislation is passed, for example, regarding headscarves. Our analysis provides a meaningful context for further exploration of cultural frames and raises important questions about the mechanisms of their economic effects. For Muslims, these questions go beyond discrimination in employment and include analysis of gender relations and, in households, intergenerational support. Future studies should, thus, be mindful of the nature and significance of these effects and their interpretation in terms of cultural framing, as opposed to matters of immigrant socioeconomic characteristics, labor market structures, or social welfare regimes.

## References

[bibr1-01979183211035725] Adida C. L. Laitin D. D. Valfort M.-A. . 2016. Why Muslim Integration Fails in Christian-Heritage Societies. Cambridge: Harvard University Press.

[bibr2-01979183211035725] Alba R. Foner N. . 2015. Strangers No More: Immigration and the Challenges of Integration in North America and Western Europe. Princeton, NJ: Princeton University Press.

[bibr3-01979183211035725] Algan Y. Dustmann C. Glitz A. Manning A. . 2010. “The Economic Situation of First and Second-Generation Immigrants in France, Germany and the United Kingdom.” The Economic Journal 120(542):F4–F30.

[bibr4-01979183211035725] Amiraux V. 2012. Muslims in Paris. Report for the at Home in Europe Project. New York, NY: Open Society Foundation.

[bibr5-01979183211035725] Attewell P. Kasinitz P. Dunn K. . 2010. “Black Canadians and Black Americans: Racial Income Inequality in Comparative Perspective.” Ethnic and Racial Studies 33(3):473–495.

[bibr6-01979183211035725] Baland J.-M. Bonjean I. Guirkinger C. Ziparo R. . 2016. “The Economic Consequences of Mutual Help in Extended Families.” Journal of Development Economics 123:38–56.

[bibr7-01979183211035725] Banting K. Kymlicka W. . 2015. “Multiculturalism Policy Index,” http://www.queensu.ca/mcp/ (accessed July 1, 2020).

[bibr8-01979183211035725] Bauböck R. 2006. Migration and Citizenship. Legal Status, Rights and Political Participation. Amsterdam: Amsterdam University Press.

[bibr9-01979183211035725] Beauchemin C. Hamel C. Simon P. . 2018. Trajectories and Origins: Survey on the Diversity of the French Population. Cham, CH: Springer, INED Population Studies.

[bibr10-01979183211035725] Blanchard N. Bancel N. Lemaire S. . 2005. La Fracture Coloniale: La Société Française au Prisme de L‘Héritage Colonial. Paris: La Découverte.

[bibr11-01979183211035725] Bloemraad I. 2012. Understanding “Canadian Exceptionalism” in Immigration and Pluralism Policy. Washington, DC: Migration Policy Institute.

[bibr12-01979183211035725] Bommes M. Geddes A. . 2000. Immigration and Welfare: Challenging the Borders of the Welfare State. London: Routledge.

[bibr13-01979183211035725] Borjas G. 1999. Heaven's Door: Immigration Policy and the American Economy. Princeton: Princeton University Press.

[bibr14-01979183211035725] Bouchard G. 2011. “What Is Interculturalism?” McGill Law Journal 562:435–468.

[bibr15-01979183211035725] Bowen J. 2007. Why the French Don’t Like Headscarves. Princeton: Princeton University Press.

[bibr16-01979183211035725] Bradbury B. Corak M. Waldfogel J. Washbrook E. . 2015. Too Many Children Left Behind: The U.S. Achievement Gap in Comparative Perspective. New York: Russell Sage.

[bibr17-01979183211035725] Brochmann G. 2015. “Immigration and the Nordic Welfare State: A Tense Companionship.” In Race, Ethnicity and Welfare States. An American Dilemma?, edited by Kettunen P. Michel S. Petersen K. , 83–106. Cheltenham: Edward Elgar Publishing.

[bibr18-01979183211035725] Chen W. Hou F. . 2019. Intergenerational Education Mobility and Labour Market Outcomes: Variation Among the Second Generation of Immigrants in Canada. Ottawa: Statistics Canada, Catalogue no. 11F0019 M — No. 418.

[bibr19-01979183211035725] Collectif Contre l'Islamophobie en France. 2019. Rapport 2019 du CCIF. Saint-Denis, France: CCIF. http://www.islamophobie.net/2019/03/15/rapport-2019-du-ccif/ (accessed July 29, 2020).

[bibr20-01979183211035725] Connor P. Koenig M. . 2013. “Bridges and Barriers: Religion and Immigrant Occupational Attainment Across Integration Contexts.” International Migration Review 47(1):3–38.

[bibr21-01979183211035725] DeVoretz D. Pivenko S. . 2005. “The Economic Causes and Consequences of Canadian Citizenship.” Journal of International Migration and Integration 6(3–4):435–468.

[bibr22-01979183211035725] Environics Institute. 2007. “Muslims and Multiculturalism in Canada.” https://www.environicsinstitute.org/docs/default-source/project-documents/survey-of-canadian-muslims/final-report.pdf?sfvrsn=af5d4536_2 (accessed July 29, 2020).

[bibr23-01979183211035725] Environics Institute. 2016. 2016 Survey of Muslims in Canada. Toronto: Environics Institute.

[bibr24-01979183211035725] Esping-Anderson G. 1990. The Three Worlds of Welfare Capitalism. Princeton: Princeton University Press.

[bibr25-01979183211035725] Ferrari G. Pailhé A. . 2017. “Transition to Adulthood in France: Do Children of Immigrants Differ From Natives?” Advances in Life Course Research 31:34–56.

[bibr26-01979183211035725] GELD (Groupe d’étude et de lutte contre les discriminations). 2000. “Une forme méconnue de discrimination et les emplois fermés aux étrangers: secteur privé, entreprises publiques, fonctions publiques.” Note No. 1, March.

[bibr27-01979183211035725] Hajjat A. Mohammed M. . 2013. Islamophobie: Comment les élites Francaises Fabriquent le “Problème Musulman.”. Paris: La Découverte.

[bibr28-01979183211035725] Heath A. F. Cheung S. Y. . 2007. Unequal Chances: Ethnic Minorities in Western Labour Markets. Oxford: Oxford University Press.

[bibr29-01979183211035725] Hennette-Vauchez S. 2017. “Is French Laïcité Still Liberal? The Republican Project Under Pressure (2004–15).” Human Rights Law Review 17(2):285–312.

[bibr30-01979183211035725] Hollifield J. 2014. “France.” In Controlling Immigration: A Global Perspective, edited by Hollifield J. Martin P. Orrenius P. . 3rd ed., 157–187. Stanford: Stanford University Press.

[bibr31-01979183211035725] Joly M. Reitz J. G. . 2018. “Emotional Stress and the Integration of Muslim Minorities in France and Canada.” International Migration Review 52(4):1111–1129.

[bibr32-01979183211035725] Joppke C. 2007. “Transformation of Immigrant Integration: Civic Integration and Antidiscrimination in the Netherlands, France, and Germany.” World Politics 59(2):243–273.

[bibr33-01979183211035725] Kazemipur A. 2014. The Muslim Question in Canada: A Story of Segmented Integration. Vancouver: UBC Press.

[bibr34-01979183211035725] Kelley N. Trebilcock M. . 2010. The Making of the Mosaic: A History of Canadian Immigration Policy. Toronto: University of Toronto Press.

[bibr35-01979183211035725] Koenig M. Mieke M. Güveli A. . 2016. “Religion and New Immigrants’ Labor Market Entry in Western Europe.” Ethnicities 16(2):213–235.

[bibr36-01979183211035725] Kogan I . 2006. “Labor Markets and Economic Incorporation Among Recent Immigrants in Europe.” Social Forces 85(2):697–721.

[bibr37-01979183211035725] Koopmans R. 2016. “Does Assimilation Work? Sociocultural Determinants of Labour Market Participation of European Muslims.” Journal of Ethnic and Migration Studies 42(2):197–216.

[bibr38-01979183211035725] Kymlicka W. 1998. Finding Our Way: Rethinking Ethnocultural Relations in Canada. Oxford: Oxford University Press.

[bibr39-01979183211035725] Laborde C. 2008. Critical Republicanism. The Hijab Controversy and Political Philosophy. Oxford: Oxford University Press.

[bibr40-01979183211035725] Lamont M. 2002. The Dignity of Working Men: Morality and the Boundaries of Race, Class, and Immigration. Cambridge: Harvard University Press.

[bibr41-01979183211035725] Lamont M. Moraes Silva G. Welburn J. S. Guetzkow J. Mizrachi N. Herzog H. Reis E. . 2016. Getting Respect: Responding to Stigma and Discrimination in the United States, Brazil, and Israel. Princeton: Princeton University Press.

[bibr42-01979183211035725] Laxer E. 2018. “‘We Are All Republicans’: Political Articulation and the Production of Nationhood in France's Face Veil Debate.” Comparative Studies in Society and History 6004:938–967.

[bibr43-01979183211035725] Laxer E. 2019. Unveiling the Nation: The Politics of Secularism in France and Québec. Montreal: McGill-Queen's University Press.

[bibr44-01979183211035725] Laxer E. Reitz J. G. Simon P. . 2020. “Muslims’ Political and Civic Incorporation in France and Canada: Testing Models of Participation.” Journal of Ethnic and Migration Studies 46(17):3677–3702.

[bibr45-01979183211035725] Lorcerie F. 2011. Muslims in Marseille. (At Home in Europe Project). New York: Open Society Foundations.

[bibr46-01979183211035725] Meer N. 2013. “Racialization and Religion: Race, Culture and Difference in the Study of Antisemitism and Islamophobia.” Ethnic and Racial Studies 36(3):385–398.

[bibr47-01979183211035725] Meurs D. Pailhé A. . 2008. “Descendantes D'immigrés en France : Une Double Vulnérabilité sur le Marché du Travail?” Travail, genre et sociétés 20(2):87–107.

[bibr48-01979183211035725] Model S. Lin L. . 2002. “The Cost of Not Being Christian: Hindus, Sikhs and Muslims in Britain and Canada.” International Migration Review 36(4):1061–1092.

[bibr49-01979183211035725] Modood T. 2013. Multiculturalism. 2nd ed. Cambridge: Polity Press.

[bibr50-01979183211035725] Nagra B. 2011. “‘Our Faith Was Also Hijacked by Those People’: Reclaiming Muslim Identity in Canada in A Post-9/11 Era.” Journal of Ethnic and Migration Studies 37(3):425–441.

[bibr51-01979183211035725] National Assembly of Québec. 2019. “Bill 21: An Act Respecting the Laicity of the State.” Québec City: Québec Official Publisher.

[bibr52-01979183211035725] OECD/ILO (Organisation for Economic Co-operation and Development/International Labour Organization). 2019. “Women at Work in G20 countries: Progress and policy action.” Paper prepared under Japan's G20 Presidency (2019). https://www.oecd.org/g20/summits/osaka/G20-Women-at-Work.pdf (accessed February 5, 2021).

[bibr53-01979183211035725] OECD (Organisation for Economic Co-operation and Development). 2012. Settling In: OECD Indicators of Immigrant Integration 2012. OECD Publishing.

[bibr54-01979183211035725] Okin S. M. 1999. Is Multiculturalism Bad for Women? Princeton: Princeton University Press.

[bibr55-01979183211035725] Pauti C . 2019. “Autorités Publiques, Laïcité et Discriminations Religieuses.” Hommes et Migrations 1324: 57–64.

[bibr56-01979183211035725] Pew Research Center. 2005. Islamic Extremism: Common Concern for Muslim and Western Publics. Washington, DC: Pew Research Center, July 14.

[bibr57-01979183211035725] Pew Research Center. 2019. Around the World, More Say Immigrants Are a Strength Than a Burden. Washington, DC: Pew Research Center, March.

[bibr58-01979183211035725] Portes A. Rumbaut R. . 2006. Immigrant America: A Portrait. 3rd ed. Berkeley: University of California Press.

[bibr59-01979183211035725] Reitz J. G. 1998. Warmth of the Welcome: The Social Causes of Economic Success for Immigrants in Different Nations and Cities. Boulder: Westview Press.

[bibr60-01979183211035725] Reitz J. G. 2011. “Pro-Immigration Canada: Social and Economic Roots of Popular Views.” IRPP Study, no. 20. Montreal: Institute for Research on Public Policy.

[bibr61-01979183211035725] Reitz J. G. 2012. “The Distinctiveness of Canadian Immigration Experience.” Patterns of Prejudice 46(5):518–538.

[bibr62-01979183211035725] Reitz J. G. 2014. “Canada: New Initiatives and Approaches to Immigration and Nation Building.” In Controlling Immigration: A Global Perspective, edited by Hollifield J. F. Martin P. L. Orrenius P. M. . 3rd ed, 88–116. Stanford: Stanford University Press.

[bibr63-01979183211035725] Reitz J. G. Frick J. R. Calabrese T. Wagner G. G. . 1999. “The Institutional Framework of Ethnic Employment Disadvantage: A Comparison of Germany and Canada.” Journal of Ethnic and Migration Studies 25(3):397–443.

[bibr64-01979183211035725] Reitz J. G. Phan M. B. Banerjee R. . 2015. “Gender Equity in Canada's Newly Growing Religious Minorities.” Ethnic and Racial Studies 38(5):681–699.

[bibr65-01979183211035725] Reitz J. G. Simon P. Laxer E. . 2017. “Muslims’ Social Inclusion and Exclusion in France, Québec, and Canada: Does National Context Matter?” Journal of Ethnic and Migration Studies 43(15):2473–2498.

[bibr66-01979183211035725] Reyes A. M. 2018. “The Economic Organization of Extended Family Households by Race or Ethnicity and Socioeconomic Status.” Journal of Marriage and Family 80:119–133. 29576657 10.1111/jomf.12445 PMC5863740

[bibr67-01979183211035725] Simon P. 2008. “The Choice of Ignorance: The Debate on Ethnic and Racial Statistics in France.” French Politics, Culture & Society 26(1):7–31.

[bibr68-01979183211035725] Simon P. Steichen E. . 2014. Slow Motion: The Labor Market Integration of New Immigrants in France. Washington, DC and Geneva: Migration Policy Institute and International Labour Office.

[bibr69-01979183211035725] Slama S. 2014. “Emplois Fermés: Une Exclusions Illégitime.” Plein droit 4(103):20–23.

[bibr70-01979183211035725] Small M. L. Harding D. J. Lamont M. . 2010. “Reconsidering Culture and Poverty.” Annals of the American Academy of Political and Social Science 629(1):6–27.

[bibr71-01979183211035725] Statistics Canada. 2005. Longitudinal Survey of Immigrants to Canada: A Portrait of Early Settlement Experiences. Ottawa: Statistics Canada, Special Surveys Division.

[bibr72-01979183211035725] Statistics Canada. 2017. National Household Survey, 2011. Public Use Microdata File: Individuals File; Study Documentation. Ottawa: Statistics Canada.

[bibr73-01979183211035725] van den Berg A. Plante C. Raïq H. Proulx C. Faustmann S. . 2017. Combating Poverty: Québec's Pursuit of a Distinctive Welfare State. Toronto: University of Toronto Press.

[bibr74-01979183211035725] van Reekum R. Duyvendak J. W. Bertossi C. . 2012. “National Models of Integration and the Crisis of Multiculturalism: A Critical Comparative Perspective.” Patterns of Prejudice 46(5):417–538.

[bibr75-01979183211035725] van Tubergen F. Maas I. Flap H. . 2004. “The Economic Incorporation of Immigrants in 18 Western Societies: Origin, Destination, and Community Effects.” American Sociological Review 69:704–727.

[bibr76-01979183211035725] Whitfield H. A. 2016. North to Bondage: Loyalist Slavery in the Maritimes. Vancouver: University of British Columbia Press.

[bibr77-01979183211035725] Wilkins-Laflamme S. 2018. “Islamophobia in Canada: Measuring the Realities of Negative Attitudes Toward Muslims and Religious Discrimination.” Canadian Review of Sociology 55(1):86–110. 29446538 10.1111/cars.12180

[bibr78-01979183211035725] Winks R. W. 1997. The Blacks in Canada: A History. 2nd ed. Montreal: McGill-Queens University Press.

[bibr79-01979183211035725] Zolberg A. R. Woon L. L. . 1999. “Why Islam Is Like Spanish: Cultural Incorporation in Europe and the United States.” Politics and Society 27(1):5–38.

